# Modulation of spatial learning and memory by cannabinoid–estrogen interaction in an AD-like cognitive impairment: The role of cannabinoid receptors and BDNF protein

**DOI:** 10.1016/j.ibneur.2026.09.002

**Published:** 2026-09-09

**Authors:** Mohammad Ali Mirshekar, Farzaneh Nadi, Hamed Fanaei, Mohadeseh Chahkandi

**Affiliations:** a Cellular and Molecular Research Center, Research Institute of Cellular and Molecular Sciences in Infectious Diseases, Zahedan University of Medical Sciences, Zahedan, Iran; b Clinical Immunology Research Center, Zahedan University of Medical Sciences, Zahedan, Iran; c Genetics of Non-Communicable Disease Research Center, Zahedan University of Medical Sciences, Zahedan, Iran; d Department of Physiology, School of Medicine, Zahedan University of Medical Sciences, Zahedan, Iran

**Keywords:** Estrogen, Marijuana, Learning, Memory, Alzheimer's disease

## Abstract

Alzheimer’s disease (AD) is an increasingly prevalent neurodegenerative disorder worldwide, with women showing a higher risk of developing the disease. The decline in steroid hormones after menopause may contribute to the increased susceptibility of women to neurodegenerative conditions. In parallel, cannabis-derived compounds have been reported to alleviate certain symptoms associated with neurological disorders. The present study was designed to investigate the effects of marijuana extract on cognitive impairment and hippocampal molecular markers in an ovariectomized AD-like rat model, and to evaluate whether co-administration with estradiol modifies these effects. The marijuana extract used in this study was characterized by HPLC and was found to contain 8.5% Δ9-THC. AD-like cognitive impairment pathology was induced by intra-hippocampal administration of Aβ25–35 in OVX rats. Animals were treated with marijuana extract (60 mg/kg/day, corresponding to approximately 5.1 mg/kg/day Δ9-THC) either alone or in combination with 17β-estradiol (1 mg/kg every 4 days) for 28 days. Cognitive performance was evaluated using the Morris water maze (MWM). In addition, hippocampal CB1/CB2 receptor expression and BDNF protein levels were measured to examine potential molecular associations. Our findings showed that chronic administration of the marijuana extract improved Aβ25–35-induced deficits in spatial learning and memory. Alterations in CB1 receptor expression and BDNF levels accompanied these behavioral effects. Notably, co-treatment with estradiol did not produce a synergistic effect, suggesting a complex interaction between cannabinoid-related and estrogen-related signaling pathways. These preclinical findings suggest that a THC-standardized marijuana extract may exert neuroprotective-like effects in an AD-like cognitive impairment model. However, because the extract was not fully phytochemically characterized and the mechanistic analyses were correlational, the results should be interpreted cautiously. Further studies are needed to clarify the underlying mechanisms and translational relevance of these findings.

## 1 Introduction

Alzheimer’s disease (AD) is a globally prevalent neurodegenerative disorder, currently afflicting approximately 50 million people worldwide(Fish et al., 2019; Monica Moore et al., 2021). While the etiology of AD involves a complex interplay between genetic and environmental factors, advanced age remains the most significant risk factor(Lane et al., 2018). Pathologically, the disease is characterized by the extracellular accumulation of amyloid-beta (Aβ) plaques and the hyperphosphorylation of microtubule-associated tau proteins, both of which are primary contributors to neurodegeneration. Despite decades of extensive research, an effective disease-modifying treatment to halt or reverse its symptomatic progression has yet to be established(Fish et al., 2019).

In recent decades, cannabis-derived extracts have gained attention as potential therapeutic candidates for neurodegenerative disorders. Evidence suggests that low-dose delta-9-tetrahydrocannabinol (THC) may reduce Aβ deposition in specific regions of the central nervous system (CNS) (Colizzi et al., 2020), while other studies have reported cognitive improvements in AD models following ultra-low-dose THC administration(Nitzan et al., 2021). Furthermore, the endocannabinoid system (ECS) has emerged as a critical regulator of the molecular processes underlying AD pathology(Kanwal et al., 2024). The expression patterns of ECS receptors, specifically CB1 and CB2, undergo significant alterations during disease progression(Nitzan et al., 2021). CB1 receptors (CB1R) are highly concentrated in brain regions associated with learning and memory and often show upregulated expression during the early stages of AD. Conversely, CB2 receptors (CB2R) are predominantly expressed in peripheral tissues and microglia, becoming more prominently involved during the later, inflammatory stages of the disease(Walker et al., 2019).

Epidemiological data indicate that the severity and prevalence of AD are disproportionately higher in women(Peterson, 2016). The precipitous decline in ovarian steroid hormones during menopause is thought to increase female susceptibility to cognitive decline and neurodegenerative processes(Jett et al., 2022). 17β-estradiol, the primary circulating estrogen, exerts multifaceted effects that can be neuroprotective or, in certain contexts, neuropathological(Gillies and McArthur, 2010; Patel et al., 2022). While some clinical reports suggest that hormone replacement therapy (HRT) in postmenopausal women may mitigate cognitive decline and improve memory in AD patients (Ali et al., 2023, Patel et al., 2022), other studies have shown inconsistent or even adverse outcomes(Lymer et al., 2024). Interestingly, a bidirectional interaction between steroid hormones and the ECS has been proposed (Gorzalka and Dang, 2012, Santoro et al., 2021). The ECS appears to influence the synthesis and secretion of ovarian hormones, while steroid hormones can modulate the activity and expression of the endocannabinoid receptor system (Santoro et al., 2021).

Brain-derived neurotrophic factor (BDNF) is essential for synaptic plasticity, neuronal survival, and the consolidation of spatial memory. Reduced BDNF expression is closely linked to Aβ toxicity, tau pathology, and hippocampal atrophy(Peng et al., 2009). Both cannabinoids and estrogens are known to modulate BDNF signaling: cannabinoids, acting through CB1/CB2 receptors, can enhance hippocampal BDNF release to rescue synaptic deficits(Khaspekov et al., 2004), while estrogen upregulates BDNF transcription via estrogen receptor (ER)-dependent pathways to promote neurogenesis (Sohrabji and Lewis, 2006). Given this convergence, the combined administration of marijuana-derived compounds and estrogen might potentially act to restore BDNF levels and reverse AD-associated memory impairments.

Despite the growing global burden of AD and the heightened risk in the female population, the interplay between the ECS and the hypothalamic-pituitary-gonadal (HPG) axis remains under-explored in the context of neurodegeneration. Therefore, the present study aimed to investigate the effects of a THC-standardized marijuana extract, administered alone or in combination with 17β-estradiol, on spatial learning and memory in an ovariectomized rat model of AD-like cognitive impairment. Specifically, this study examined the potential involvement of cannabinoid receptors and BDNF signaling, as an initial effort to explore whether estradiol co-administration modifies the cognitive effects of marijuana extract in a model of postmenopausal AD-like cognitive impairment.

## 2 Experimental procedures

### 2.1 Animals

Forty-nine adult female Wistar rats (8–10 weeks old) were obtained from the Animal Center of Zahedan University of Medical Sciences, Iran. The animals were housed under standard laboratory conditions at a controlled temperature of 24 ± 1 °C with a 12-h light/dark cycle and were provided free access to standard rodent chow and water. Rats were maintained under a 12:12 h light/dark cycle (lights on at 07:00 and off at 19:00). All behavioral and experimental procedures were conducted during the light phase, between 09:00 and 14:00. All experimental procedures were conducted in accordance with the ethical guidelines approved by the Animal Ethics Committee of Zahedan University of Medical Sciences (IR.ZAUMS.AEC.1401.003). All efforts were made to minimize the number of animals used and to reduce animal suffering throughout the experimental procedures.

### 2.2 Determination of the estrous cycle

To minimize variability associated with endogenous hormonal fluctuations, female rats were housed in proximity for approximately two weeks before the start of the study to facilitate estrous cycle synchronization. Vaginal smear analysis was then performed as a confirmatory measure to assess estrous stage before the experimental procedures. Vaginal smears were collected daily between 7:00 and 8:00 a.m. and stained using the Papanicolaou method. Briefly, the specimens were processed through a graded series of alcohol concentrations, stained with Eosin Azure 50 (EA50), Orange G 6 (OG6), and Harris hematoxylin, dehydrated again through graded alcohol solutions, and cleared in xylene. The estrous cycle stage was determined using a light microscope at 40 × magnification. The estrus phase was identified based on the predominance of cornified epithelial cells (Juybari et al., 2020). Based on reproductive status, to ensure a synchronized hormonal profile, all animals in the intact group were specifically selected during the estrus phase of the estrous cycle, as confirmed by vaginal cytology. The remaining animals underwent bilateral ovariectomy.

### 2.3 Bilateral ovariectomy procedures

Rats were anesthetized with a ketamine/xylazine mixture (80/10 mg/kg, intraperitoneally). Under sterile conditions, bilateral dorsal incisions were made on the back of each rat. For ovariectomy, the ovaries were identified, and the associated blood vessels were clamped, ligated, and subsequently removed along with the ovaries. The abdominal cavity was then rinsed with 1–2 mL of sterile saline, and the muscle and skin layers were sutured. In the sham-operated group, the same surgical procedure was performed, including bilateral dorsal incisions and tissue manipulation; however, the ovaries were left intact (Khaksari et al., 2015). Ovariectomy was performed two weeks after Aβ25–35 surgery, and animals were allowed a further two-week recovery period before treatment.

### 2.4 Drugs

Ketamine and xylazine were purchased from Alfasan Inc. (Utrecht, Netherlands). Amyloid β-protein fragment 25–35 (Aβ25–35) was obtained from Kimia Pajouh Dorsa (Chowdhury et al.). Marijuana plant material was provided by the Police Headquarters for Combating Narcotics in Kerman Province, Iran. 17β-estradiol (E2) and saline were obtained from Aburaihan Pharmaceutical Co. (Tehran, Iran). The BDNF ELISA kit was purchased from ZellBio GmbH (Germany) through Padginteb, Iran. Total RNA extraction and cDNA synthesis kits were purchased from Pars Tous Biotechnology Co. (Chowdhury et al.). Primers were synthesized by Gen Fanavaran Ltd. (Tehran, Iran).

### 2.5 Preparation of Marijuana extract

Marijuana plant material was provided by the Police Headquarters for Combating Narcotics in Kerman Province, Iran. Fresh leaves were air-dried at room temperature and mechanically ground into a fine powder. Briefly, 100 g of the powdered material was macerated in 1000 mL of 80% methanol (v/v; Merck, Germany) for 72 h with intermittent agitation. The extract was then filtered, and the solvent was removed under reduced pressure using a rotary evaporator. The concentrated crude extract was dried and stored in glass vials at −20 °C until use. The crude marijuana extract was standardized according to its Δ9-tetrahydrocannabinol (Δ9-THC) content using high-performance liquid chromatography (HPLC). HPLC analysis showed that the extract contained 8.5% Δ9-THC. Accordingly, the administered dose of 60 mg/kg/day of total extract corresponded to approximately 5.1 mg/kg/day Δ9-THC. It should be noted that the extract was not comprehensively phytochemically characterized for other cannabinoids, such as cannabidiol (CBD), cannabinol (CBN), minor cannabinoids, terpenes, or other plant-derived constituents. Therefore, the administered preparation is referred to throughout the manuscript as a THC-standardized marijuana extract rather than purified THC. On the day of administration, the required amount of extract was freshly prepared in 5% dimethyl sulfoxide (DMSO) and diluted to the appropriate concentration for intraperitoneal (i.p.) injection. The same vehicle was administered to the corresponding control groups.(Chahkandi et al., 2022; Motamedi et al., 2021).

### 2.6 AD-like cognitive impairment model induction in rats

Rats were anesthetized with a ketamine/xylazine mixture (80/10 mg/kg, intraperitoneally). The Aβ25–35-induced AD-like cognitive impairment model was established by bilateral intra-hippocampal injection using a stereotaxic apparatus. Briefly, Aβ25–35 was dissolved in sterile saline and injected bilaterally into hippocampus at a dose of 1.5 mg/kg in a volume of 5 μL. The stereotaxic coordinates were as follows: 4.16 mm posterior to bregma, ±2.5 mm lateral to the sagittal suture, and 2.6 mm below the skull surface. A 26-gauge needle was slowly inserted into each lateral ventricle through a small burr hole drilled in the skull. Sham-operated animals underwent the same surgical procedure; however, an equal volume of sterile saline was injected instead of Aβ25–35.(Kim et al., 2014).

### 2.7 Experimental groups and drug administration

**Diagram 1:**
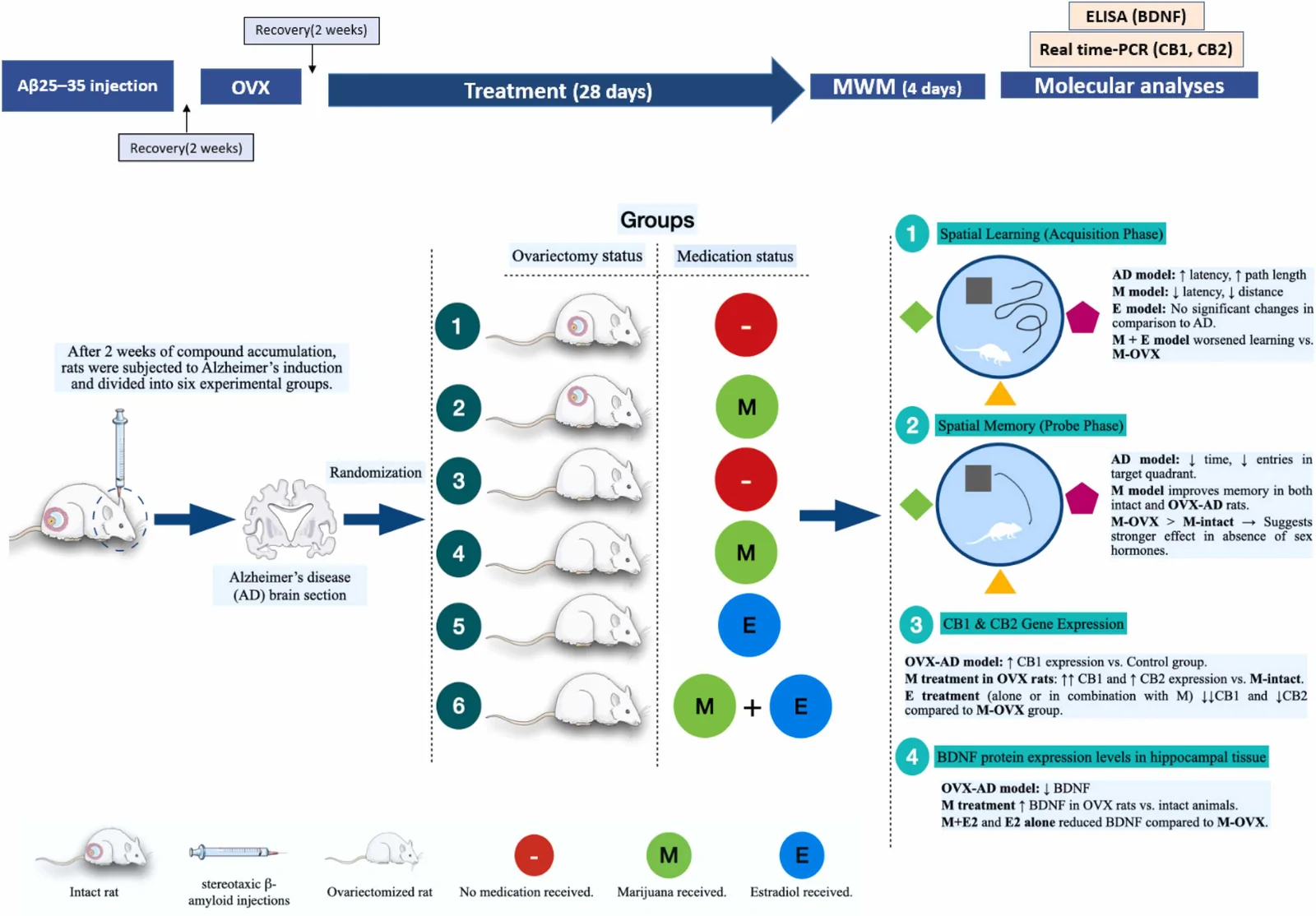
Schematic timeline of the experimental design. The protocol began with intra-hippocampal Aβ25–35 microinjection to induce an AD-like model, followed by a 2-week recovery period. Bilateral ovariectomy (OVX) was performed on day 14. After an additional 2-week recovery period to allow endogenous estrogen depletion, pharmacological treatments (marijuana extract, 17β-estradiol, or their co-administration) were initiated on day 28 and continued for 28 days. Morris Water Maze (MWM) testing was conducted during the final 4 days of treatment, after which hippocampal tissues were collected for molecular analyses, including ELISA for BDNF and RT-PCR for CB1 and CB2.

To confirm successful induction of the AD-like cognitive impairment model, sham-operated rats without AD-like cognitive impairment (n = 7) that received sterile saline instead of Aβ25–35 were included for comparison in the Morris water maze test. Following Aβ25–35 injection, rats with AD-like cognitive impairment were randomly assigned to six experimental groups (n ~7 per group): Ctr, AD-like rats with intact ovaries that received vehicle; M, intact rats with AD-like cognitive impairment receiving THC-standardized marijuana extract; Ctr-OVX, ovariectomized rats with AD-like cognitive impairment receiving vehicle; M-OVX, ovariectomized rats with AD-like cognitive impairment receiving THC-standardized marijuana extract; E2-OVX, ovariectomized rats with AD-like cognitive impairment receiving 17β-estradiol; and M + E2-OVX, ovariectomized rats with AD-like cognitive impairment receiving both THC-standardized marijuana extract and 17β-estradiol. The experimental design and methodology are summarized in Diagram 1. The THC-standardized marijuana extract was administered intraperitoneally at 60 mg/kg/day of total extract for 28 consecutive days, corresponding to approximately 5.1 mg/kg/day Δ9-THC. 17β-estradiol was administered intraperitoneally at 1 mg/kg every four days for 28 days. Treatment solutions were prepared with a final DMSO concentration of 5% when applicable, and control animals received the corresponding vehicle according to the same injection schedule and volume as their respective treatment groups. Potential biological effects of DMSO cannot be completely excluded and are acknowledged as a limitation.

The experimental timeline was as follows: rats first underwent stereotaxic bilateral intra-hippocampal injection of Aβ25–35, followed by a 2-week recovery period. Ovariectomy was then performed in the designated groups, followed by an additional 2-week recovery period before initiation of treatments. Behavioral testing was conducted after treatment completion, and tissue collection was performed after the behavioral assessments.

### 2.8 Morris water maze (MWM) test

Spatial learning and memory were assessed using the Morris water maze (MWM) test, a widely used behavioral paradigm for evaluating hippocampus-dependent spatial navigation in rodents. The Morris water maze consisted of a circular pool measuring 150 cm in diameter and 50 cm in height, filled with water to a depth of 30 cm and maintained at 22 ± 1°C. A hidden platform (10 cm diameter) was submerged 1.5 cm below the water surface. Because the platform was not visible, rats were required to locate it using extra-maze visual cues. The MWM protocol was conducted over four consecutive days. During the acquisition phase (days 1–3), each rat received three trials per day from randomized starting positions, none of which was located in the platform quadrant. Each trial was terminated when the rat found the hidden platform or after a maximum duration of 60 s. Rats that failed to locate the platform within 60 s were gently guided to it and allowed to remain on the platform for 10–15 s. Escape latency, path length, and swimming speed were automatically recorded during each trial. On day 4, memory retention was evaluated in a 60-s probe trial, during which the platform was removed. The percentage of time spent in the former platform quadrant and the number of crossings over the previous platform location were recorded using an automated video-tracking system (Haghparast et al., 2018). To rule out the potential confounding effect of treatment- or surgery-induced motor deficits on spatial learning parameters, the average swim speed (cm/s) of each animal was also recorded and analyzed across all training sessions.

### 2.9 Molecular analysis by quantitative real-time PCR

At the end of the experimental protocol, rats were deeply anesthetized with a mixture of ketamine 10% (75 mg/kg, i.p.) and xylazine 2% (10 mg/kg, i.p.). The hippocampi were rapidly isolated using a standardized procedure. After completion of the behavioral tests, rats were deeply anesthetized, and the hippocampi were rapidly dissected. One hippocampus from each animal was immediately placed in RNA preservation solution and stored at −20 °C for subsequent CB1 and CB2 receptor mRNA expression analysis by quantitative real-time PCR. The contralateral hippocampus was stored at −80 °C until determination of BDNF protein levels by ELISA. Total RNA was extracted using an RNA extraction kit (Parstous, Iran) according to the manufacturer’s instructions. The purity and concentration of the extracted RNA were determined using a NanoDrop 2000 spectrophotometer (Thermo Fisher Scientific, USA). Complementary DNA (cDNA) was synthesized from total RNA using a cDNA synthesis kit (Parstous, Iran). Quantitative real-time PCR was performed under the following cycling conditions: initial denaturation at 94 °C for 5 min, followed by 40 cycles of denaturation at 94 °C for 30 s, annealing at 58 °C for 30 s, and extension at 72 °C for 30 s. β-actin was used as the internal reference gene for normalization. The primers were synthesized by Gen Fanavaran Ltd. (Tehran, Iran), and their sequences are listed in Table 1. All reactions were performed at least in triplicate. Relative mRNA expression levels were calculated using the comparative Cq method (2^−ΔΔCq), with β-actin used as the internal reference gene.

**Table 1:** Gene primer sequences.

Gene	Forward sequence	Reverse sequence
*CB1*	*ACTGGTGCTGTGTGTCATCC*	*GTCCACATCAGGCAAAAGGC*
*CB2*	*AAGAAGAAGCCCCAAAGTCCT*	*AGAGATGTTTGCTGGGTGGC*
*β-Actin*	*CGC AGT GGA AGT CAG GCT TG*	*GGC ATA GCG TAC ACA GCG TC*

### 2.10 *ELISA* analysis for *BDNF* level

Hippocampal BDNF protein levels were measured using a rat-specific ELISA kit (ZellBio, Germany; Cat. No. ZB-10476C-R9648), according to the manufacturer’s instructions. After completion of behavioral testing, animals were deeply anesthetized, and hippocampal samples were collected, rapidly dissected on ice, and immediately frozen in liquid nitrogen. The hippocampal tissue was cut into small pieces and homogenized in ice-cold PBS. Total protein was extracted using freshly prepared lysis buffer supplemented with protease inhibitors, including 1 mM phenylmethylsulfonyl fluoride, 2.5 μg/mL leupeptin, 10 μg/mL aprotinin, and 1 mM sodium orthovanadate, using a tissue homogenizer (Silent Crusher S, Germany). The homogenates were centrifuged at 6000 rpm for 15 min at 4 °C. The supernatants were collected and assayed according to the manufacturer’s instructions. Absorbance was measured at 450 nm using a microplate reader (Model 680 Microplate Reader, Bio-Rad Laboratories, CA, USA), and BDNF concentrations were calculated based on the corresponding standard curve. BDNF concentrations were normalized to total protein content and expressed as pg/mg protein (Haghparast et al., 2018).

## 3 Statistical analysis

Statistical analysis was performed using IBM SPSS Statistics (version 26). Normality of the data was assessed using the Shapiro–Wilk test. To confirm successful induction of AD-like cognitive impairment, the sham and Aβ25–35 groups were compared using an independent-samples *t*-test. For the Morris water maze (MWM) acquisition phase, behavioral data were analyzed using a three-way repeated-measures ANOVA, with OVX and treatment as between-subjects factors and training day as the within-subjects factor. Greenhouse–Geisser correction was applied when the assumption of sphericity was violated. Probe trial and gene expression data were analyzed using two-way ANOVA, with OVX and treatment as fixed factors, followed by Tukey’s post hoc test for multiple comparisons. Effect sizes were reported as partial eta squared (ηp²). All data are presented as mean ± SEM, and statistical significance was set at p < 0.05.

## 4 Results

### 4.1 Spatial learning phase

**Fig. 1:**
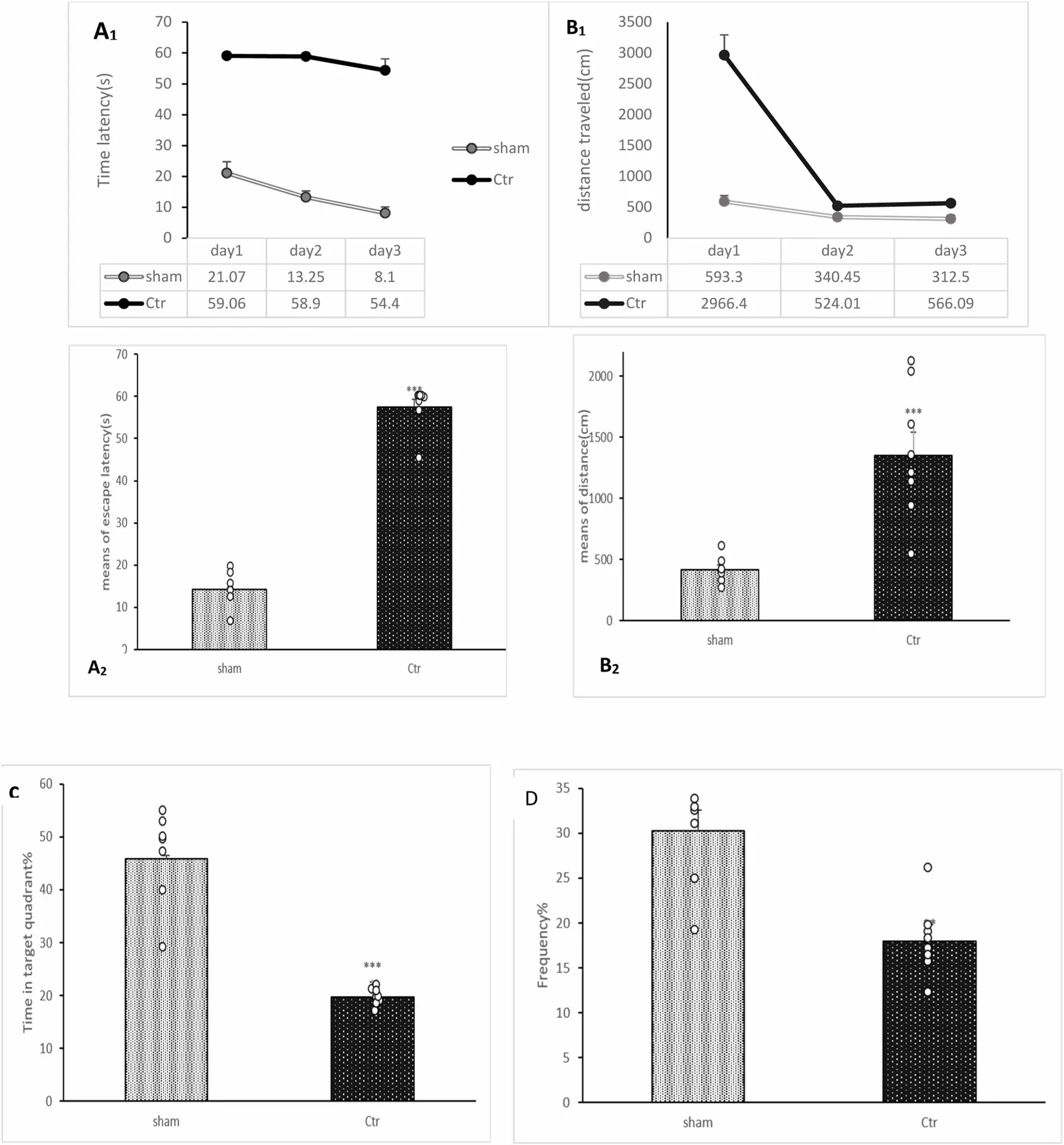
Morris water maze performance in sham-operated rats and AD-like model control rats with intact ovaries. The sham group consisted of sham-operated rats with intact ovaries that received sterile saline instead of Aβ25–35. The Ctr group consisted of Aβ25–35-injected rats with intact ovaries that received vehicle and served as the AD-like model control group. (A_1,2_) Escape latency to locate the hidden platform during the acquisition phase. (B_1,2_) Path length (distance traveled) to reach the hidden platform during the acquisition phase. (C) Time spent in the target quadrant during the probe trial. (D) Number of crossings over the former platform location during the probe trial. Data are presented as mean ± SEM. **p < 0.01 and *p < 0.001 versus the sham group. AD-like, Aβ25–35-induced Alzheimer’s disease-like cognitive impairment; Ctr, Aβ25–35-injected.

Spatial learning was assessed using two parameters: path length, defined as the distance traveled to reach the hidden platform, and escape latency, defined as the time required to locate the platform. To confirm successful induction of AD-like cognitive impairment, the sham and Aβ25–35 groups were compared during the three-day acquisition phase of the Morris water maze (Fig. 1A1, B1). For escape latency, a two-way mixed repeated-measures ANOVA with training day as the within-subject factor and group as the between-subject factor revealed a significant main effect of training day, *F (1.44, 18.68) = 7.33, p = 0.008, ηp2 = 0.361* and a significant main effect of group, *F (1, 13) = 311.87, p < 0.001, ηp2 = 0.960*, whereas the day × group interaction was not significant, *F (1.44, 18.68) = 2.03, p = 0.168, ηp2= 0.135*. For path length to reach the hidden platform, Mauchly’s test indicated violation of the sphericity assumption *(W = 0.055, p < 0.001);* therefore, Greenhouse–Geisser-corrected results are reported. There were significant main effects of training day, *F (1.028, 13.367) = 22.881, p < 0.001, ηp2 = 0.638* and group, *F (1, 13) = 21.180, p < 0.001, ηp2 = 0.620*, as well as a significant training day × group interaction, *F (1.028, 13.367) = 14.715, p = 0.002, ηp2 = 0.531*. The Aβ25–35 group traveled a greater distance to locate the hidden platform than the sham group, and the pattern of change across training days differed significantly between the two groups (Fig. 1B1). Overall, the Aβ25–35 group showed longer escape latency and path length than the sham group across the acquisition days. To provide an overall summary of acquisition performance, the mean values across the three training days were compared between groups using independent-samples *t*-tests (Fig. 1A2, B2). These analyses showed that Aβ25–35 significantly worsened escape latency*)* $t\left(13\right)=17.66$*,* p<0.001*)* and path length *(t (7.67) = 4.92, p = 0.001)* relative to sham controls.

**Fig. 2:**
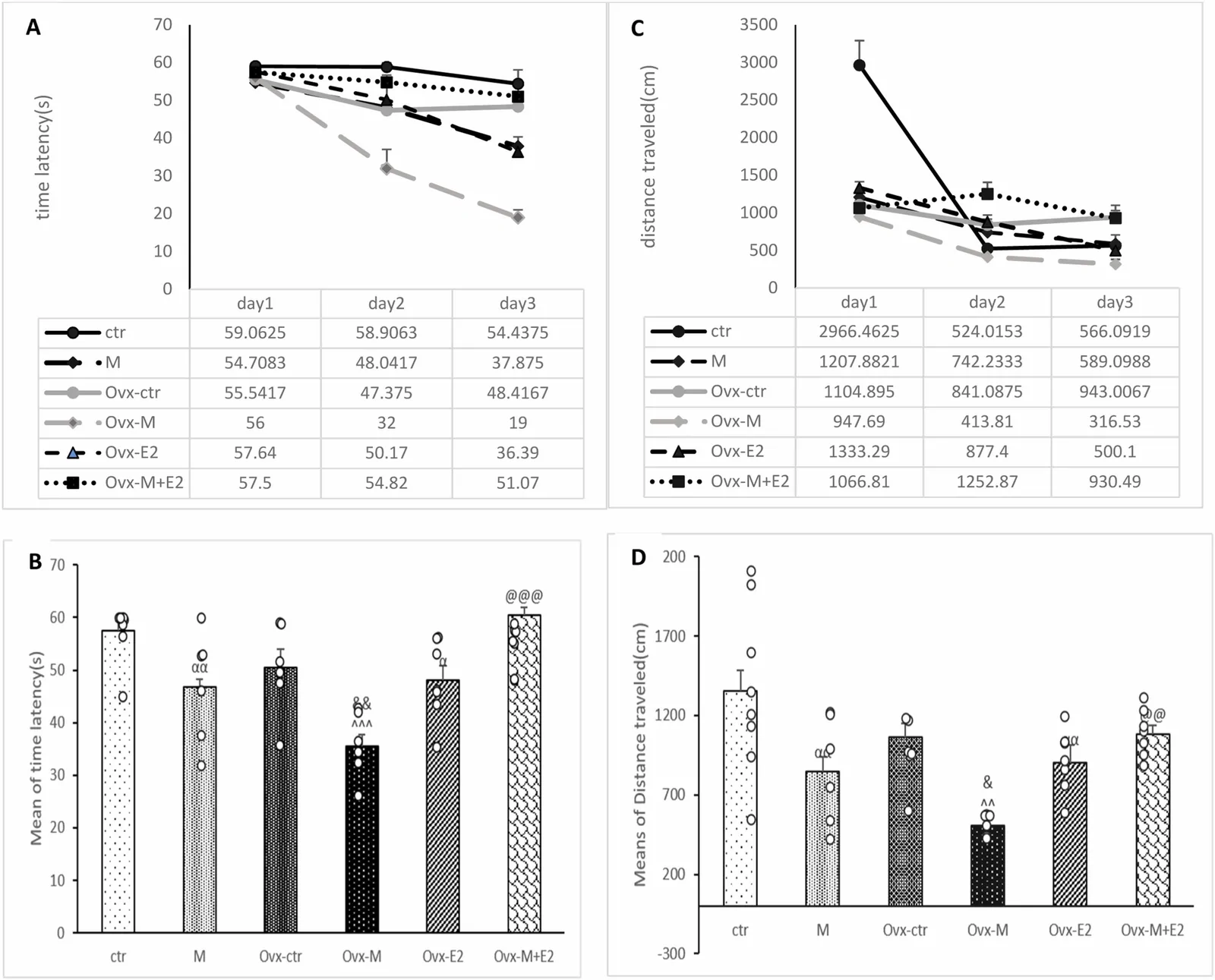
The effects of marijuana and estrogen co-treatment on spatial learning in the MWM test and comparison of studied groups. (A and B) Comparison of the escape latency in studied groups (C and D) Comparison of the distance traveled in study groups. Data are shown as mean ± SEM (*n* ~ 7). ^*&*^*p < 0.05,*^& &^*p* < 0.01 for M-OVX vs. M, ^*αα*^*p* < 0.01*,*^α^*p* < 0.05 for M and E2-OVX vs. Ctr*,*^*^^*^*p* < 0.01, ^^^^^*p* < 0.001 for M-OVX vs. Ctr-OVX, ^*@@@*^*p* < 0.001 for M + E2-OVX vs. M-OVX. Ctr (control), E2 (17β-estradiol), M (marijuana), OVX (ovariectomized rat).

As shown in Fig. 2, the overall analysis of all experimental groups using three-way repeated-measures ANOVA for escape latency showed a significant main effect of day, *F (2, 68) = 50.53, p < 0.001, ηp² = 0.598.* Mauchly’s test indicated that the assumption of sphericity was not violated (*W = 0.930, p = 0.304).* The day × OVX interaction was not significant*, F (2, 68) = 0.39, p = 0.676, ηp² = 0.011,* whereas the day × treatment interaction was significant, *F (6, 68) = 6.96, p < 0.001, ηp² = 0.380*. Notably, the day × OVX × treatment interaction was also significant, *F (2, 68) = 7.48, p = 0.001, ηp² = 0.180.* Between-subjects analysis showed no significant main effect of OVX, *F (1, 34) = 0.11, p = 0.745, ηp² = 0.003,* but significant effects of treatment, *F (3, 34) = 7.10, p = 0.001, ηp² = 0.385,* and the OVX × treatment interaction, *F (1, 34) = 9.49, p = 0.004, ηp² = 0.218*. Three-way repeated-measures ANOVA for the distance traveled to reach the hidden platform during MWM acquisition showed a significant main effect of day, *F (1.21, 40.97) = 38.47, p < 0.001, ηp² = 0.531.* Mauchly’s test indicated that the assumption of sphericity was violated *(W = 0.340, p < 0.001);* therefore, Greenhouse-Geisser-corrected results were reported. The day × OVX interaction was significant, *F (1.21, 40.97) = 14.99, p < 0.001, ηp² = 0.306,* and the day × treatment interaction was also significant, *F (3.62, 40.97) = 6.23, p = 0.001, ηp² = 0.355.* Notably, the day × OVX × treatment interaction was significant as well, *F (1.21, 40.97) = 3.94, p = 0.047, ηp² = 0.104*. Between-subjects analysis showed no significant main effect of OVX, *F (1, 34) = 0.84, p = 0.367, ηp² = 0.024*, but significant effects of treatment, *F (3, 34) = 3.68, p = 0.021, ηp² = 0.245,* and the OVX × treatment interaction, *F (1, 34) = 8.79, p = 0.006, ηp² = 0.205*.

To further explore these interactions, post hoc comparisons (Tukey’s HSD) were performed on the mean performance across the three training days (Fig. 2B, and D). Results showed that the M-OVX group had significantly lower escape latency and path length than the M group (p < 0.01 and p < 0.05, respectively), suggesting that marijuana extract (equivalent to 5.1 mg/kg THC) exerted a more pronounced positive effect on learning in the absence of sex hormones. Furthermore, the M group exhibited significantly better acquisition performance than the Ctr group *(p < 0.01)*. In OVX rats, M administration reduced escape latency and path length compared with the Ctr-OVX group *(p < 0.001 and p < 0.01, respectively).* Interestingly, concurrent administration of M + E2 in rats increased escape latency and path length compared with the M-OVX group *(p < 0.001),* suggesting a possible interaction between cannabinoids and estrogen during the acquisition phase. Compared with the AD-like Cognitive Impairment control (Ctr) group, the E2-OVX group exhibited significantly reduced escape latency and path length to find the hidden *platform (p < 0.05 and p < 0.01, respectively)*. To ensure that changes in escape latency were not biased by differences in locomotor ability, the mean swim speed was analyzed. No significant differences in average swim speed were observed among the experimental groups during the training days (all p>0.05; data not shown), indicating that the treatments and surgical procedures did not induce motor impairments affecting swimming performance.

### 4.2 Spatial memory phase (Probe Test)

**Fig. 3:**
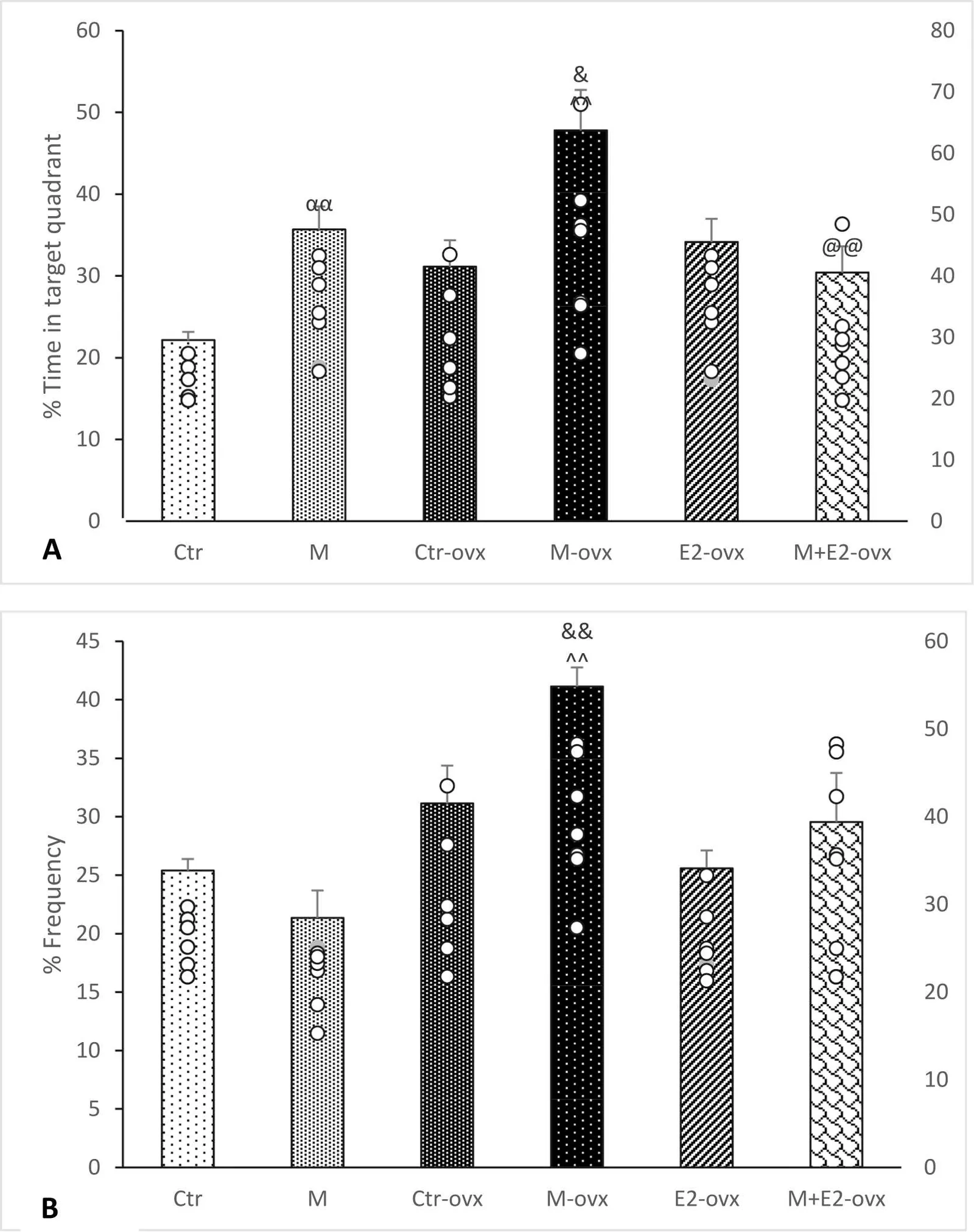
The effects of marijuana and estrogen co-treatment on spatial memory in the MWM test and comparison of study groups. (A) Comparison of time spent in the target quadrant studied groups (B) Comparison of frequency in the target quadrant in studied groups. Data are shown as mean ± SEM (*n* = 7). ^& &^*p* < 0.01, ^&^*p* < 0.05 for M-OVX vs. M, ^αα^*p* < 0.01 for M vs. Ctr^, ^^^*p* < 0.01 for M-OVX vs. Ctr-OVX^, @@^*p* < 0.01 for M + E2-OVX vs. M-OVX. Ctr (control), E2 (17β-estradiol), M (marijuana), OVX (ovariectomized rat).

In the probe trial (Fig. 1C, D), independent-samples *t*-tests were used to compare memory-retention indices between the sham and Aβ25–35 groups, revealing significant impairment in the Aβ25–35 group (*t (7.59) = 8.60, p < 0.001)* and the frequency of crossings over the target location) *t (10.27) = 4.52, p = 0.001).* The experimental groups were then analyzed separately using two-way ANOVA followed by Tukey’s post hoc test. Two-way ANOVA for the percentage of time spent in the target quadrant during the Morris water maze probe trial showed no significant main effect of OVX, *F (1, 34) = 1.27, p = 0.268, ηp² = 0.036*. However, there was a significant main effect of treatment, *F (3, 34) = 7.17, p = 0.001, ηp² = 0.388.* In addition, the OVX × treatment interaction was significant, *F (1, 34) = 8.33, p = 0.007, ηp² = 0.197,* indicating that the effect of treatment on target quadrant performance differed by OVX status. Post hoc analysis showed that M treatment significantly improved spatial memory in intact animals compared with the Ctr group *(p < 0.01).* In ovariectomized rats, the M-OVX group spent more time in the target quadrant and showed a higher crossing frequency than the M-intact group *(p < 0.05 and p < 0.01, respectively).* In addition, a significant improvement was observed in the M-OVX group compared with the Ctr-OVX group *(p < 0.01).* However, co-administration of E2 and M in the M + E2-OVX group significantly reduced the time spent in the target quadrant compared with the M-OVX group *(p < 0.01),* suggesting that E2 may interfere with the memory-enhancing effects of the marijuana extract in this model (Fig. 3).

### 4.3 *CB1* and *CB2* gene expression levels in hippocampal tissue

**Fig. 4:**
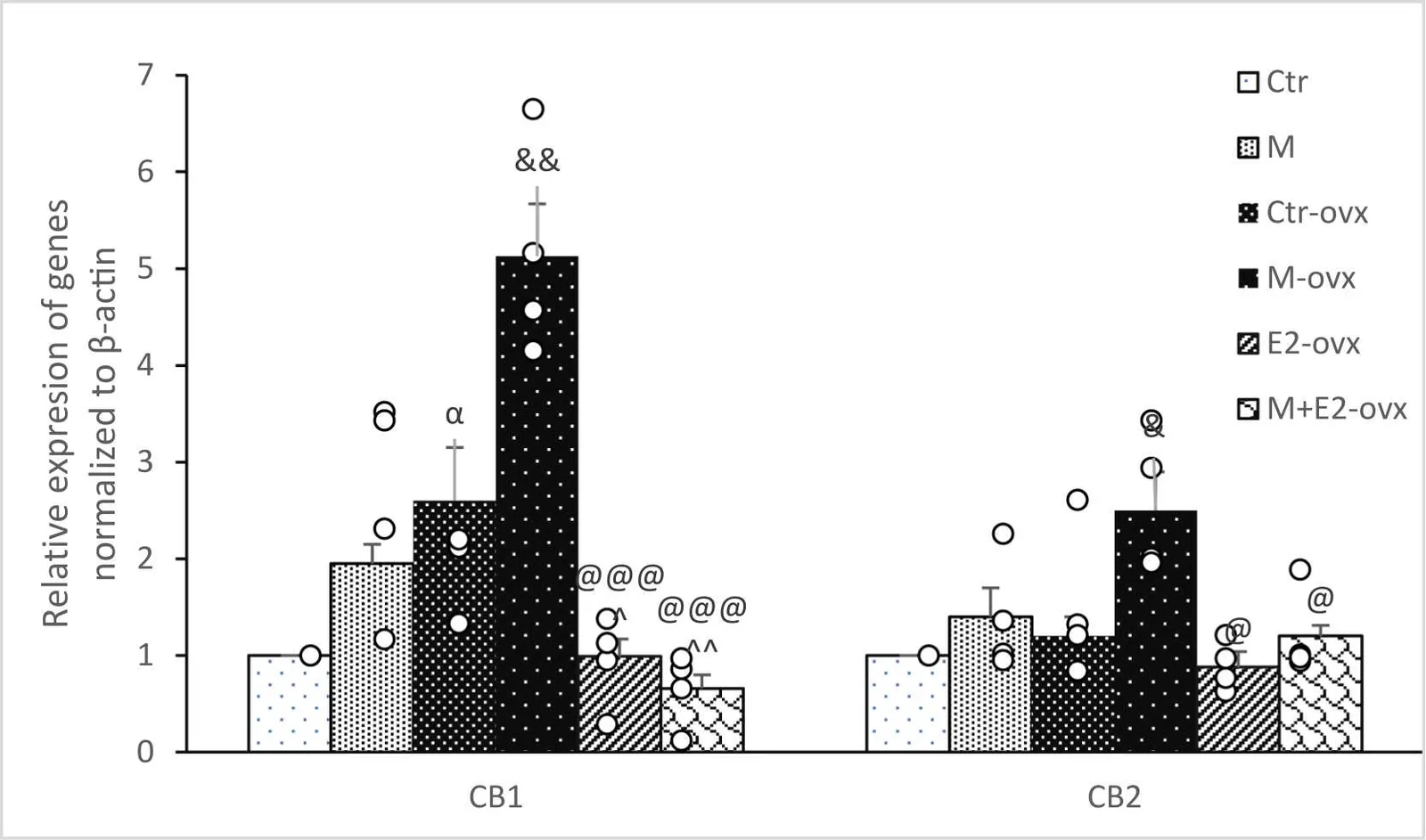
The effects of marijuana and estrogen co-treatment on the concentration of *CB1* and *CB2* genes in the hippocampus and comparison of studied groups. Data are shown as mean ± SEM (*n* = 7). ^α^ p < 0.05 for Ctr-OVX vs Ctr. ^*&*^*p* < 0.05, ^*& &*^*p* < 0.001 for M-OVX vs. M, ^@^*p* < 0.05, ^*@@@*^*p* < 0.001 for M + E2^-^OVX vs. M-OVX, ^^^ p < 0.05, ^*^^*^*p* < 0.01 for E2-OVX and M + E2-OVX vs. Ctr-OVX. Ctr (control), E2 (17β-estradiol), M (marijuana), OVX (ovariectomized rat).

Fig. 4 shows the mRNA expression levels of the CB1 and CB2 genes. Two-way ANOVA showed significant main effects of OVX and treatment on *CB1* gene expression *[OVX: F (1, 18) = 46.62, p < 0.001, ηp² = 0.721;* treatment*: F (3, 18) = 33.96, p < 0.001, ηp² = 0.850]*. A significant OVX × treatment interaction was also observed for *CB1*, *F (1, 18) = 5.03, p = 0.038, ηp² = 0.218.* For *CB2* gene expression, both OVX and treatment also exerted significant main effects *[OVX: F (1, 18) = 7.59, p = 0.013, ηp² = 0.297;* treatment: *F (3, 18) = 7.50, p = 0.002, ηp² = 0.556],* whereas the OVX × treatment interaction was not significant, *F (1, 18) = 1.64, p = 0.217, ηp² = 0.084.* The expression levels of these genes were normalized to β-actin and analyzed using two-way ANOVA, followed by Tukey’s post hoc test where appropriate. The ovariectomized rats with AD-like cognitive impairment exhibit an apparent gain in *CB1 mRNA* copy number compared to the Ctr group (*p* < 0.05) (Fig. 4). In contrast, the comparison of these groups shows no clear difference in the level of *CB2* expression. Administration of marijuana in OVX animals increases *CB1* (*p* < 0.01) and *CB2* (*p* < 0.05) *mRNA* levels compared with the M in the intact group. The copy number of *CB1 and CB2 mRNAs* is lower in the M + E2-OVX *(p < 0.001, and p < 0.05)* and E2-OVX *(p < 0.001, and p < 0.05)* groups compared to the M-OVX group.

### 4.4 *BDNF* protein contents in hippocampal tissue

**Fig. 5:**
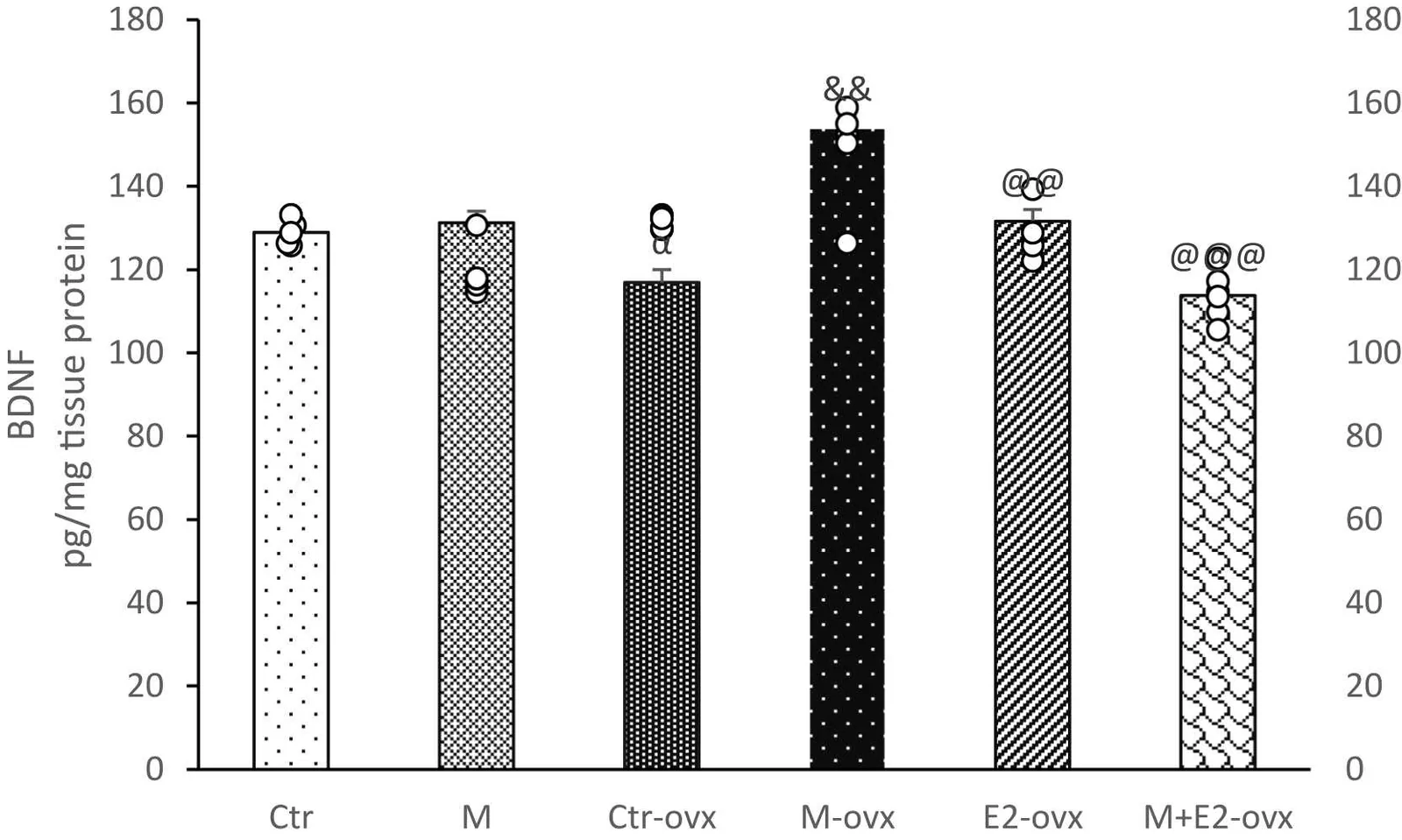
The hippocampal concentration of *BDNF* in the experimental groups. (A) The *BDNF* protein levels in the hippocampus were measured using the ELISA method. Data are shown as mean ± SEM (*n* = 7). ^α^*p* < 0.05 for Ctr-OVX vs Ctr. ^& &^*p* < 0.01 for M-OVX vs. M, ^*@@*^*p* < 0.01, ^*@@@*^*p* < 0.001 for E2-OVX and M + E2-OVX vs. M-OVX. Ctr (control), E2 (17β-estradiol), M (marijuana), OVX (ovariectomized rat).

Two-way ANOVA for *BDNF* levels showed a significant main effect of OVX, *F (1, 23) = 6.65, p = 0.017, ηp² = 0.224*, as well as a significant main effect of treatment*, F (3, 23) = 58.15, p < 0.001, ηp² = 0.884*. Moreover, the OVX × treatment interaction was significant*, F (1, 23) = 73.24, p < 0.001, ηp² = 0.761.* The comparison of the *BDNF* concentration in the treatment group is presented in Fig. 5. The BDNF protein content decreases in the Ctr-OVX group compared to the Ctr group (*p* < 0.05); furthermore, a clear decrease is observed between the M + E2-OVX and E2-OVX groups and the M-OVX group (*p* < 0.001 and *p < 0.01).* As shown in Fig. 5, the M administration increases *BDNF* protein levels in the ovariectomized rats relative to intact animals (*p* < 0.01).

## 5 Discussion

The present study investigated the effects of a THC-standardized marijuana extract and 17β-estradiol (E2), either alone or in combination, on spatial learning and memory as well as hippocampal molecular markers, including CB1 and BDNF, in a model of AD-like cognitive impairment. The main findings can be summarized as follows. First, chronic administration of the marijuana extract improved spatial learning and memory retention in Aβ-injected rats, and these behavioral effects were accompanied by increased hippocampal CB1 mRNA expression and BDNF protein levels. Second, under the current dosing regimen, E2 treatment did not significantly affect performance in the acquisition or probe trials and did not substantially alter CB1 mRNA expression or BDNF levels compared with the Ctr-OVX group. Third, co-administration of marijuana extract and E2 appeared to attenuate the beneficial effects observed with marijuana extract alone, particularly with respect to spatial learning, CB1 mRNA expression, and BDNF concentrations in the ovariectomized AD-like cognitive impairment model.

Numerous studies have investigated the effects of cannabinoids on learning and memory. Our previous study (2022) examined the effects of marijuana and estrogen on spatial memory in aged animals(Chahkandi et al., 2022), and the present study extends those findings to an AD-like cognitive impairment model. Interestingly, the findings of both studies were consistent regarding the effects of marijuana. The present data suggest that cannabinoid constituents, including THC, may be associated with modulation of cognitive performance and changes in cannabinoid receptor-related markers(Colizzi et al., 2020). Previous reports have shown that CB1 receptor deletion impairs memory and exerts age-dependent effects on spatial learning (Albayram et al., 2012).]. Although CB2 receptors have been associated with Aβ processing, CB2 appears to play a secondary role compared with CB1 in mediating cognitive behaviors and therapeutic responses (Aso et al., 2016, Niloy et al., 2023). Consistently, Kim et al. (2023) reported that THC and CBD reduced Aβ, phosphorylated tau, and calcium levels and improved cognitive performance in AD-related in vivo and in vitro models (Kim et al., 2023). However, cannabinoid effects on cognition are dose-dependent and may vary across experimental conditions. Some studies have suggested that THC may impair learning through CB1- and BDNF-mediated mechanisms depending on the dose(Niloy et al., 2023). In the present study, although the administered extract dose was 60 mg/kg/day, the estimated THC exposure was approximately 5.1 mg/kg/day. Therefore, the pharmacological relevance of this dose should be interpreted cautiously. Rodent cannabinoid dosing cannot be directly translated to human cannabis exposure because of species-specific differences in metabolism, bioavailability, route of administration, and pharmacodynamic sensitivity. Thus, these findings should be considered preclinical and hypothesis-generating and should not be directly extrapolated to clinical use without further dose-response, pharmacokinetic, and safety studies.

Previous work from our laboratory showed that ovariectomy impairs learning and memory, which provided part of the rationale for including estrogen-deficient animals in the present study. The effects of estradiol on cognition are also heterogeneous. While several studies suggest that estrogen positively affects learning and memory (Cooke and Woolley, 2005, Gillies and McArthur, 2010); other reports have indicated potential risks associated with estradiol therapy in certain populations (Ali et al., 2023). Conversely, Pourhadi et al. (2023) evaluated the risk of dementia with estradiol therapy and found an increased risk in certain populations (Pourhadi et al., 2023). In the present study, estrogen treatment for 28 days, administered every 4 days to mimic the estrous cycle, did not significantly affect spatial learning in the AD-like cognitive impairment model. Moreover, co-administration of estradiol with the THC-standardized marijuana extract did not enhance the behavioral or molecular effects of the extract. The attenuated response observed in the co-treatment group may reflect a complex interaction between estrogenic and endocannabinoid signaling under the specific dose, timing, and OVX conditions used in this study. Consistent with our findings, some meta-analyses have failed to demonstrate a definitive association between AD and estrogen therapy (Song et al., 2020). AD-related reductions in estrogen receptor number and affinity may contribute to a diminished response to hormone therapy (Ali et al., 2023). In addition, progesterone may potentially block the neuroprotective pathways of estrogen (Ali et al., 2023). Since BDNF signaling is considered one of the mechanisms underlying estrogen action, the absence of BDNF modulation in the E2-treated group is consistent with the behavioral findings (Song et al., 2020).

When looking at the combined administration of the marijuana extract and 17β-estradiol, our behavioral assays did not reveal any synergistic or additive improvements in MWM performance compared to either treatment alone. A likely explanation for this is a physiological ceiling effect in spatial learning, where each treatment on its own might have already pushed cognitive recovery to its maximum potential within this Alzheimer’s model. Alternatively, this lack of synergy could point to shared downstream signaling pathways in the hippocampus. Both estradiol and cannabinoid systems are known to influence hippocampal plasticity through partially overlapping molecular cascades (Chowdhury et al., 2024), reflecting the well-documented bidirectional relationship between the endocannabinoid system and gonadal hormones (López, 2010). Although we did not map these intracellular pathways directly, the potential saturation of these common signaling hubs offers a plausible mechanism for the lack of additive effects. This complexity is further highlighted by the receptor-level crosstalk between the two systems; for instance, estradiol can modulate CB1 receptor density in a region-specific manner(Hill et al., 2007). The interplay between the endocannabinoid system (ECS) and estrogen signaling (ES) is clearly multifaceted. While our data suggest a minor role for the CB2 receptor—likely due to its lower density in the central nervous system (Santoro et al., 2021). Because estradiol modulates CB1 expression differently depending on the brain region, the specific interactions observed here are likely unique to the hippocampal microenvironment and the doses we selected.

Since our study focused on overall therapeutic outcomes rather than dissecting these detailed molecular pathways, these findings should be interpreted with caution. Future studies utilizing varying dose ratios and selective pathway blockers will be essential to determine whether this lack of additive effect stems from receptor-level antagonism or simply a natural limit of hippocampal functional recovery. Finally, safety remains a critical consideration when evaluating cannabinoid-based therapies. While targeting the endocannabinoid system holds promise for neurodegenerative diseases, cannabinoid treatments—especially those containing THC—can cause adverse cognitive, emotional, motor, and physiological side effects depending on the dose, duration, age, sex, and exact chemical composition (Testai et al., 2022). Chronic or high-dose THC exposure has been linked to impaired learning and memory, altered anxiety levels, locomotor changes, and neuroendocrine disruptions. Consequently, the cognitive benefits observed in our preclinical model must be carefully balanced against these potential risks, underscoring the need for further research to map the therapeutic window and long-term safety profile of THC-standardized extracts.(Safi et al., 2024; Testai et al., 2022).

Notwithstanding the positive outcomes observed, several limitations of the present study should be acknowledged. First, the marijuana extract was standardized primarily based on its THC content; thus, a comprehensive phytochemical characterization of other cannabinoids, terpenes, and bioactive constituents was not performed. Consequently, the observed effects should be attributed to the whole-plant extract rather than exclusively to THC or isolated receptor activation. Second, while the extract dose corresponded to approximately 5.1 mg/kg of THC, the translational relevance of this regimen is constrained by species-specific differences, the intraperitoneal route of administration, and the absence of detailed pharmacokinetic or long-term safety assessments.

Furthermore, the mechanistic interpretation of our findings is limited by the lack of receptor antagonist experiments, CB1/CB2 protein-level validation, and direct downstream signaling analyses. Similarly, since only a single dose and administration schedule of 17β-estradiol were evaluated, it remains possible that alternative dosing regimens could yield different therapeutic interactions. It should also be noted that the Aβ25–35 model reflects acute amyloid toxicity and may not fully capture the chronic, multifactorial pathology of human Alzheimer’s disease. Finally, due to practical and financial constraints, vehicle groups were not included in the molecular assays. While our behavioral data showed no significant differences between the vehicle and control groups, the absence of these controls in the molecular analysis represents a limitation in fully ruling out potential vehicle-specific effects on protein expression. These factors should be taken into account when interpreting the behavioral and molecular findings of this study.

## 6 Conclusion

In conclusion, chronic administration of THC-standardized marijuana extract improved spatial learning and memory performance in the AD-like cognitive impairment model, and these behavioral changes were accompanied by alterations in hippocampal CB1/CB2 gene expression and BDNF protein levels. Estradiol co-treatment did not produce a clear synergistic effect under the present experimental conditions. However, because the study used a crude extract standardized only for THC, an acute amyloid toxicity model, and correlative molecular endpoints, the findings should be interpreted cautiously. Further studies using fully characterized cannabinoid preparations, multiple doses, receptor-specific pharmacological tools, protein-level validation, and chronic AD models are required to clarify the mechanisms and translational relevance of these observations.

## CRediT authorship contribution statement

**Mohammad Ali Mirshekar:** Writing – review & editing, Supervision, Methodology, Investigation. **Farzaneh Nadi:** Resources, Project administration, Methodology. **Hamed Fanaei:** Software, Formal analysis, Data curation. **Mohadeseh Chahkandi:** Writing – original draft, Supervision, Investigation, Conceptualization.

## Ethics declaration

This study was conducted in accordance with the ARRIVE (Animal Research: Reporting of In Vivo Experiments) guidelines. This study was approved by the Animal Ethics Committee of Zahedan University of Medical Sciences. (Approval No. IR.ZAUMS.AEC.1401.003)

## Declaration of Competing Interest

The authors declare that they have no known competing financial interests or personal relationships that could have appeared to influence the work reported in this paper.
